# Compressibility of High-Density EEG Signals in Stroke Patients

**DOI:** 10.3390/s18124107

**Published:** 2018-11-23

**Authors:** Nadia Mammone, Simona De Salvo, Cosimo Ieracitano, Silvia Marino, Emanuele Cartella, Alessia Bramanti, Roberto Giorgianni, Francesco C. Morabito

**Affiliations:** 1 IRCCS Centro Neurolesi Bonino Pulejo, Via Palermo c/da Casazza, SS. 113, 98124 Messina, Italy; simona.desalvo@irccsme.it (S.D.S.); silvia.marino@irccsme.it (S.M.); emanuele.cartella@irccsme.it (E.C.); roberto.giorgianni@irccsme.it (R.G.); 2 DICEAM Department, *Mediterranea* University of Reggio Calabria, Via Graziella Feo di Vito, 89060 Reggio Calabria, Italy; cosimo.ieracitano@unirc.it (C.I.); morabito@unirc.it (F.C.M.); 3 Institute of Applied Sciences and Intelligent Systems *Eduardo Caianiello* (ISASI), National Research Council (CNR), Via Torre Bianca, Mortelle, Istituto Marino, 98164 Messina, Italy; alessia.bramanti@irccsme.it

**Keywords:** compressive sensing, High-Density Electroencephalography, stroke

## Abstract

Stroke is a critical event that causes the disruption of neural connections. There is increasing evidence that the brain tries to reorganize itself and to replace the damaged circuits, by establishing compensatory pathways. Intra- and extra-cellular currents are involved in the communication between neurons and the macroscopic effects of such currents can be detected at the scalp through electroencephalographic (EEG) sensors. EEG can be used to study the lesions in the brain indirectly, by studying their effects on the brain electrical activity. The primary goal of the present work was to investigate possible asymmetries in the activity of the two hemispheres, in the case one of them is affected by a lesion due to stroke. In particular, the compressibility of High-Density-EEG (HD-EEG) recorded at the two hemispheres was investigated since the presence of the lesion is expected to impact on the regularity of EEG signals. The secondary objective was to evaluate if standard low density EEG is able to provide such information. Eighteen patients with unilateral stroke were recruited and underwent HD-EEG recording. Each EEG signal was compressively sensed, using Block Sparse Bayesian Learning, at increasing compression rate. The two hemispheres showed significant differences in the compressibility of EEG. Signals acquired at the electrode locations of the affected hemisphere showed a better reconstruction quality, quantified by the Structural SIMilarity index (SSIM), than the EEG signals recorded at the healthy hemisphere (*p* < 0.05), for each compression rate value. The presence of the lesion seems to induce an increased regularity in the electrical activity of the brain, thus an increased compressibility.

## 1. Introduction

**Figure 1:**
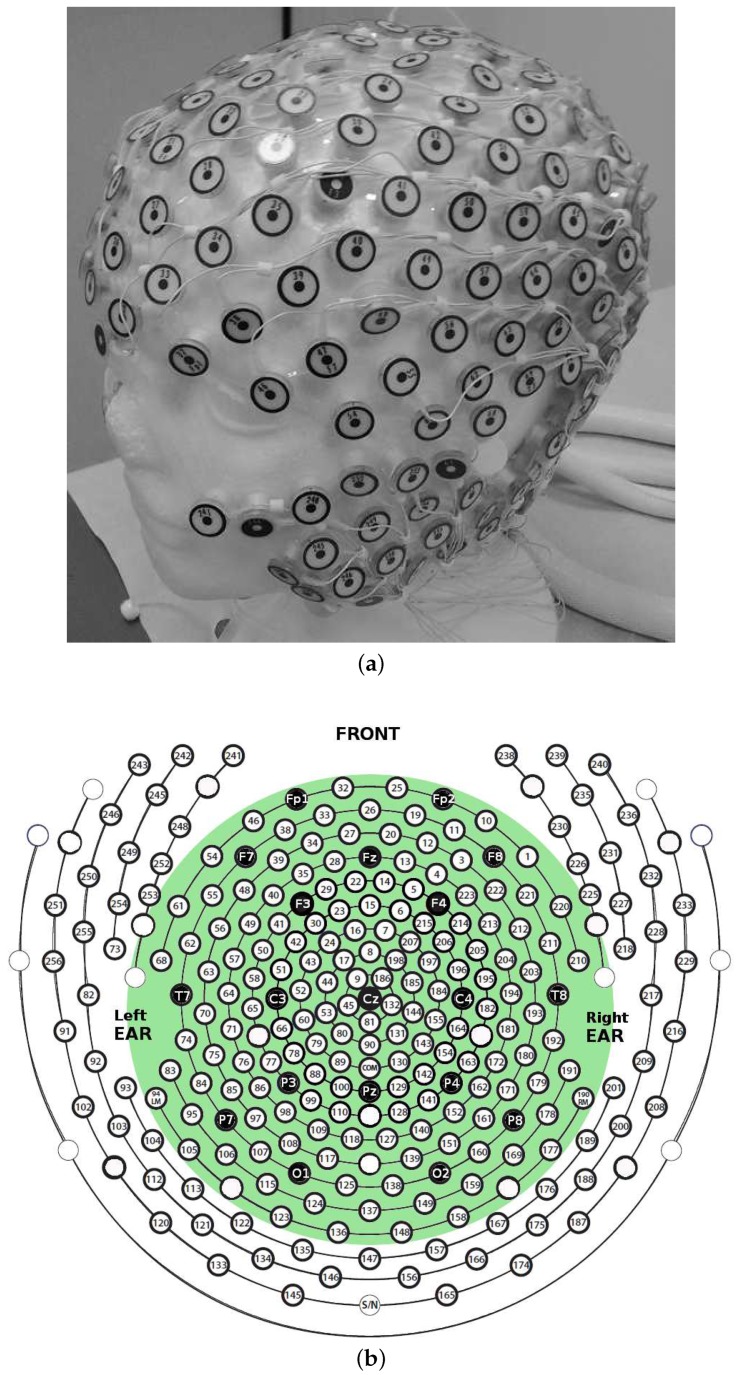
EEG recording system: (**a**) The 256-channel High-Density *Electrical Geodesics EEG system*. (**b**) A 2D representation of the 256-channel High-Density electrode montage. The selected 162 scalp electrodes are those enclosed in the green area. Black electrodes represent the standard low-density 19-channel montage.

Electroencephalography (EEG) is a neurophysiological technique that collects at the scalp the electrical potentials produced by the bio-electromagnetic fields generated by the intra- and extra-cellular current flows related to neuronal activity. EEG is widely used in neurology because many neurological disorders cause an impairment in the electrical activity of the brain [1,2,3,4,5]. EEG offers a very high temporal resolution together with a poor spatial resolution, either because of volume conduction effects [6] or because of the large inter-electrode distance that characterizes standard 10/20 EEG montage (represented with black electrodes in Figure 1b), which is 7 cm on average. It ia demonstrated that an inter-sensor distance of 1–2 cm would allow to improve the spatial resolution of EEG [7]. With High-Density 256-channel EEG (HD-EEG), which comprises all the electrodes shown in Figure 1b, a very good spatial resolution can be achieved [7].

Stroke is a critical event that can be hemorrhagic, in the case of a blood vessel disruption, or ischemic, in the case the brain is deprived of oxygen and other components.

There is increasing evidence that cerebral stroke causes the disruption of neuronal networks [8,9]. Soon after, the brain works to rewire and/or to establish novel alternative communication pathways to compensate for lost or damaged circuits, both locally or remotely to the lesion [10]. Therapy and rehabilitation aim at aiding the brain to restore its functionality but finding the optimal treatment is still a challenging task that can take weeks. Moreover, since the ability of the brain to recover seems to decay over time, there is a great deal of interest in finding objective biomarkers of functional recovery that can be computed non-invasively and safely many times over the short term. Such biomarkers would be estimated from proper neurophysiological measurements of the patient and could be used as measures of functional recovery throughout the patient’s follow-up program. Among all the possible neurophysiological measurements, EEG seems to be the best candidate that such biomarkers could be based on [11,12,13]. EEG is indeed totally non-invasive and generally well-tolerated by patients. EEG is also a relatively cheap and fast examination. In recent years, researchers have employed EEG to study brain’s functionality after stroke. Zeng et al. [14] quantified the effects of stroke rehabilitation by estimating the Mean NonLinearly Separable complexity Degree (Mean NLSD) of EEG signals. They introduced Mean NLSD as a possible indicator for the evaluation of stroke rehabilitation effect. They applied it to 32-channel EEGs recorded from a cohort of 11 stroke patients and 11 healthy controls. A decrease in Mean NLSD could be observed in almost all the analyzed scalp electrodes at lesion locations. Caliandro et al. [15] investigated whether acute stage ischemic stroke affected the organization of cortical networks. Thirty unilaterally impaired acute stroke patients were studied together with 30 healthy controls. Their resting state EEGs were recorded through 19 channels. Changes in resting state network parameters (small worldness) were mainly observed in delta and high-alpha bands in both hemispheres, in either right- or left-lesioned patients. Liu et al. [16] explored the nonlinear features of EEG-based functional connectivity, in patients with acute thalamic ischemic stroke and healthy subjects. Nineteen-channel resting state EEGs were recorded from 12 stroke patients and 11 healthy subjects. Lempel–Ziv complexity (LZC), Sample Entropy (SampEn) and partial directed coherence (PDC) were calculated. A higher EEG complexity and a weaker functional connectivity were detected in the stroke patients, whereas the stroke group exhibited a lower SampEn than the control group in alpha band. Zappasodi et al. [17] assessed the relationship between Fractal Dimension (FD) and clinical impairment and between FD and recovery prognosis in acute stroke. Nineteen-channel resting state EEGs were collected in 36 patients 4–10 days after a unilateral ischemic stroke and 19 healthy controls. FD resulted smaller in patients than in controls and its reduction was shown to be correlated to a worse acute clinical status. The loss of complexity was hypothesized to be related to the global system dysfunction. All the above-mentioned studies are based on standard low-density EEG. Only Zeng et al. [14] and Caliandro et al. [15] compared the features of the EEGs collected at the impaired zones with the those recorded at the healthy zones: Caliandro et al. [15] observed no difference between the two hemispheres of the same subject, whereas Zeng et al. [14] claimed that the method they proposed needed to be further validated on EEG time series. As regards high-density EEG studies, De Vico Fallani et al. [18] applied source imaging to HD-EEG to evaluate the compensatory reorganization of brain networks after cerebellar damage during a finger extension task. Sixty-four-channel EEGs were acquired during alternating movement tasks. Patients were compared to healthy controls and exhibited significant topological differences in their brain networks. One of the issues that comes with EEG recording, especially with HD-EEG, is the size of the recorded files. EEG files are always meant for storage (because they are reviewed offline by experts for diagnostic purposes) over the long term (because a patient is usually periodically evaluated and her/his examinations are compared longitudinally). EEG datasets are also often meant to be shared with other research centers, thus they need to be transmitted. This is the reason EEG signal compression has drawn much attention from researchers recently [19,20,21,22].

Compressibility in EEG signal analysis is mainly meant for reducing the size of EEG signals to optimize storage and transmission. However, studies on the compressibility of pathological EEGs showed that the quality of EEG signal reconstruction reflected alterations in the electrical activity of the brain, such as in Alzheimer’s Disease (AD) [23], and could therefore be relevant to diagnosis. In [23], AD is shown to be associated with an altered compressibility of EEG signals; in particular, AD patients showed a higher compressibility, compared to healthy subjects, probably due to the inherent increased regularity of EEG signals due to cortical atrophy [24]. In the present research, the compressibility of EEG signals acquired at the two hemispheres of unilaterally impaired stroke patients was investigated. It was hypothesized that the two hemispheres exhibited different compressibility characteristics because of the effects of lesions on the interaction between neurons. To this purpose, HD-EEG signals recorded from 18 stroke patients, who were unilaterally impaired, were compressed and reconstructed by means of Block Sparse Bayesian Learning (BSBL) [19]. When the 256-channel high electrode density configuration was used, the two hemispheres exhibited significantly different reconstruction quality characteristics (*p* < 0.05). It is worth highlighting that such differences were lost when only the electrodes belonging to the standard low-density 19-channel configuration were included the analysis. The paper is organized as follows. Section 2.2 illustrates how patients were enrolled in the study and how their HD-EEGs were recorded and preprocessed. Section 2.4 introduces how EEG signals were compressively sensed and how the reconstruction quality of the EEG recorded at the two hemispheres was estimated. Section 3 reports the achieved results. Section 4 and Section 5 draw some conclusions and address future perspectives.

## 2. Methodology

### 2.1. Patients’ Description

Eighteen stroke patients were enrolled and monitored at the rehabilitative post-stroke unit of IRCCS Centro Neurolesi Bonino Pulejo (Messina, Italy). A multidisciplinary team of neurologists, neuropsychologists and EEG experts planned and conducted all the medical examinations. The study was carried out according to the guidelines of a clinical protocol approved by the local Ethics Committee (Approved N. 003/17).The subjects and their caregivers were informed about the purposes of the present study that were also reported in an informed consent form that was signed by the participants and/or their caregivers. Patients with previous ischemic or hemorrhagic stroke were excluded. Patients with history of other neurological diseases, traumatic brain injury, defects in sight, previous depression or other psychiatric disorders were also excluded. Every patient underwent a neuroradiological examination to localize the lesion site and the patients exhibiting lesions in both hemispheres were excluded. Table 1 reports age (mean age 65.67 ± 15.14), gender, severity scale (scored according to the NIH Stroke Scale [25]), stroke type (ischemic or hemorrhagic), the diagnosis of the stroke event (stroke site) and the impaired hemisphere (lesion site). The recordings were acquired 3–6 months after the event. All patients received antiplatelet therapy (10 aspirin and 8 clopidogrel), 12/18 assumed antihypertensive treatment and 10/18 statin therapy. The EEGs were recorded in the morning. The patients were fully clinically evaluated and reported detailed information about the last night sleep and the last meal before the EEG recording session started.

### 2.2. HD-EEG Recording and Preprocessing

EEG signals were acquired by means of a high-density 256-channel *EGI Sensor Net*, which comes with the *Electrical Geodesics EEG system* (Figure 1a). The sampling rate was 250 Hz. Every channel *x* acquires the differential potential between *x* and the reference electrode (central sensor Cz). Following EGI guidelines, electrode impedance was kept <50 kΩ. The HD-EEG sensor net was placed as depicted in Figure 1a. Figure 1b shows the high-density channels montage. The low-density 19-channel montage includes only the black electrodes. The subjects kept their eyes closed but remained awake (*eye-closed resting state*) during EEG acquisition. EEG signals were bandpass-filtered between 1 and 40 Hz by the *Net Station EEG software*, which comes with the *Electrical Geodesics EEG system*, to include the major EEG waves: delta (1–4 Hz), theta (4–8 Hz), alpha (8–13 Hz), beta (13–30 Hz), and gamma (>30 Hz). Once filtered, the recordings were manually reviewed by the EEG experts to mark and discard the artifactual segments. In particular, two EEG experts reviewed the recordings independently and marked the epochs contaminated by blinking spikes, ocular movements, muscular artifacts and any other possible kind of artifact. In the future, automatic artifact rejection methods based on Independent Component Analysis [26] will be taken into account. Four minutes of artifact-free HD-EEG were finally selected for each patient. The electrodes located over the cheeks and neck were not included in the analysis, because they are heavily contaminated by muscle and bad contact artifacts. In the end, 162 out of the 256 available channels were considered (enclosed in the green area in Figure 1b). The pre-processed HD-EEG signals were then exported as Matlab *.mat* files to be further processed by means of compressive sensing, as described in Section 2.4. All algorithms were developed in Matlab 2016a (The MathWorks, Inc., Natick, MA, USA).

### 2.3. Compressive Sensing

*Compressive Sensing* (CS) technique is based on the assumption that *sparse* signals can be recovered by using a small number of significant measurements. Specifically, a signal **x** $\in R^{Mx1}$ with *C* non-zero coefficients (C<<M), and consequently M−C zero values, is called C−sparse. A sparse signal can be represented by a projection of suitable orthonormal basis:

(1) x=Ψs

where **x** denotes the signal $\in R^{Mx1}$, Ψ is the orthonormal basis matrix $\in R^{MxM}$ and **s** is the vector of sparse coefficients $\in R^{Mx1}$. The formulation of CS problem is commonly expressed as follows:

(2) y=Φx

where **y** is the compressed representation $\in R^{Nx1}$ (with N < M), **x** is the sparse signal $\in R^{Mx1}$, and Φ is denoted as *measurement matrix* $\in R^{NxM}$. As x=Ψs (Equation (1)), the compressed representation can be expressed as:

(3) y=ΦΨs=Θs

where Θ=ΦΨ represents the *sensing matrix*. The CS algorithm reconstructs **s** by using **y** and Θ (Equation (3)), and then, recovers the original signal **x** by using Ψ and **s** (Equation (1)), [27,28,29].

However, CS technique is applicable only to a subset of physiological signals. In this context, although EEG signals do not have sparsity properties in the time or in the transformed domain, CS paradigm can be adopted by applying the Block Sparse Bayesian Learning (BSBL) technique proposed by Zhang et al. [20]. The BSBL algorithm assumes that the original signal *x* can be split into *m* non-overlapping blocks, where only a few of them are non-zeros. The *i*th block is modeled as a parameterized multivariate Gaussian distribution:

(4) $$p\left(x_{i};\varsigma_{i},B_{i}\right)=N\left(x_{i};0,\varsigma_{i}B_{i}\right),\;\mathrm{with}\;i=1,...,m$$

where $\varsigma_{i}$ is a nonnegative parameter introduced to control the block-sparsity of *x* and $B_{i}$ is a positive definite matrix introduced to capture the intra-block correlation. The algorithm assumes the independence between blocks and also that the noise vector satisfies a multivariate Gaussian distribution, p(v)=N(0,λΓ) where λ and Γ indicate a positive scalar and the identity matrix, respectively. Based on the assumptions of the previous probabilistic model, the evaluation of *x* can be achieved through the maximum a posteriori estimation. Detailed mathematical formulation of BSBL is reported in [19]. Therefore, in this study, the BSBL algorithm was employed to recover the HD-EEG signal according to the compressive sensing formulation. A real-world application of CS is for example telemonitoring. The HD-EEG (x) is firstly compressed (y) and then transmitted to a remote expert, who recovers the original HD-EEG signals according to Equation (3) [19]. The quality of the reconstruction may be estimated by the Structural SIMilarity index (SSIM) [19]. SSIM measures the similarity index between the original signal and the recovered one and shows better recovery performance than Mean Squared Error (MSE), especially for structured signals. SSIM = 1 indicates perfect recovery of the original signal. Specifically, SSIM was introduced by Wang et al. [30] as follows: let *x* and *y* be two signals to be compared (the original signal and the reconstructed one); the SSIM index is defined as follows:

(5) $$SSIM\left(x,y\right)=\frac{2\mu_{x}\mu_{y}+C_{1}}{\mu_{x}^{2}\mu_{y}^{2}+C_{1}}\cdot \frac{2\sigma_{x}\sigma_{y}+C_{2}}{\sigma_{x}^{2}\sigma_{y}^{2}+C_{2}}\cdot \frac{\sigma_{xy}+C_{3}}{\sigma_{x}\sigma_{y}+C_{3}}$$

where $\mu_{x}$ and $\sigma_{x}$ denote the mean and the standard deviation of *x*, respectively; $\mu_{y}$ and $\sigma_{y}$, similarly, denote mean and standard deviation of *y*, respectively; and $\sigma_{xy}$ represents the cross correlation between *x* and *y*. C_1_, C_2_, and C_3_ are small positive constants values, introduced to guarantee numerical stability of aforementioned statistical parameters ($\mu_{x}$, $\mu_{y}$, $\sigma_{x}$, $\sigma_{y}$, $\sigma_{xy}$). C_1_, C_2_, and C_3_ were set as 0 in the present work [31]. Detailed mathematical formulation of SSIM is reported in [30].

### 2.4. Compression of EEG Signals

**Figure 2:**
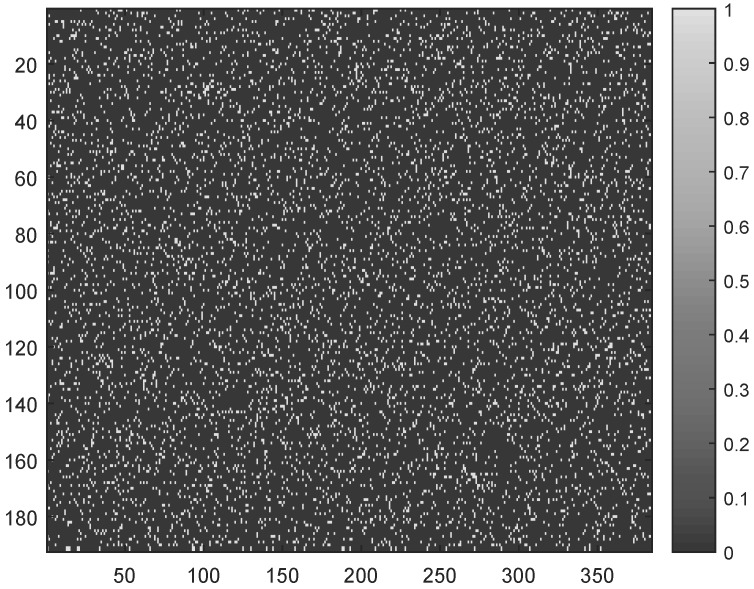
Example of sparse binary sensing matrix sized 192 × 384, corresponding to a 50% compression rate. Black elements are zero, white elements are 1.

The main objective of the present work was to study the asymmetry in the cerebral electrical activity of the two hemispheres of patients with unilateral stroke. The hypothesis was that the presence of a lesion alters the compressibility of EEG signals recorded at the impaired hemisphere. The secondary objective was to assess if the hypothesized differences are more prominent when the analysis is based on HD-EEG rather than on standard low-density EEG montage. To this purpose, a group of 18 patients was enrolled as described in Section 2.2 and underwent eye-closed resting state HD-EEG recording. Four minutes (4 × 60 = 240 s) of artifact-free recording were selected for each patient (with a f_*s*_ = 250 Hz sampling rate) and preprocessed as described in Section 2.2. Specifically, each EEG signal was split into *m* non-overlapping epochs of 1.536 s length (as f_*s*_ = 250 Hz, the EEG epoch *e* included *M* = 1.536 × 250 = 384 data samples), for a grand total of *m* = 240/1.536 = 156 epochs. In the adopted BSBL approach, simple binary sparse Φ matrices of size *N* × *M* were used (where *M* = 384 indicates the EEG epoch length and *N* depends on the compression rate, defined as CR = $\frac{M-N}{M}$ × 100). For example, CR = 50% means setting N = 192. An explanatory sensing matrix (sized 192 × 384, thus providing a 50% compression rate) is shown in Figure 2.

The 162 selected electrodes (see Section 2.2) were equally divided into two sub-sets, corresponding to the right and left hemispheres. For every epoch *e*, every EEG signal *x* in the two subsets was compressed and then reconstructed at an increasing Compression Rate (from 50% to 90% with a 5% step) and the related quality of reconstruction was estimated as $SSIM^{e}\left(x\right)$.

#### 2.4.1. Epoch-Based SSIM Comparison

To quantitatively compare the SSIM of the two hemispheres, for every patient and for every epoch *e*, the SSIM values $SSIM^{e}{\left(x\right)}_{R}$ of the channels of the right hemisphere and the SSIM values $SSIM^{e}{\left(x\right)}_{L}$ of the channels of the left hemisphere were averaged over the channels. In this way, a vector of 156 elements (as many as the analyzed epochs) for the right hemisphere ${\mathrm{SSIM}}_{R}$ and a vector of the same size for the left hemisphere ${\mathrm{SSIM}}_{L}$ were obtained. To compare the SSIM of the two hemispheres, the SSIM values of the right hemisphere ($SSIM^{e}{\left(x\right)}_{R}$) and the SSIM values of the left hemisphere ($SSIM^{e}{\left(x\right)}_{L}$) were averaged over the channels. In this way, a vector of 156 elements (one element per epoch) for the right hemisphere ${\mathrm{SSIM}}_{R}$ was obtained. The vector ${\mathrm{SSIM}}_{L}$ of the left hemisphere was obtained in the same way. The Shapiro–Wilk test [32] showed that the two populations, ${\mathrm{SSIM}}_{R}$ and ${\mathrm{SSIM}}_{L}$, were not normally distributed and the Mann–Whitney test [33] was used to compare their medians. The hypothesis was that the SSIM of the impaired hemisphere is significantly higher than the SSIM of the preserved hemisphere, for every CR. The results were not corrected for multiple comparisons.

#### 2.4.2. Overall SSIM Comparison

For each patient, the mean SSIM of the two hemispheres, averaged over the epochs, was also compared. In particular, the values $SSIM^{e}{\left(x\right)}_{R}$ and $SSIM^{e}{\left(x\right)}_{L}$ were averaged over the channels *x* and over the epochs *e*, thus obtaining the average ${\bar{SSIM}}_{R}$ resulting from the reconstruction of the EEG signals of the right hemisphere and of the left one ${\bar{SSIM}}_{L}$, for increasing CR values (from 50% to 90% with a 5% step).

## 3. Results

**Figure 3:**
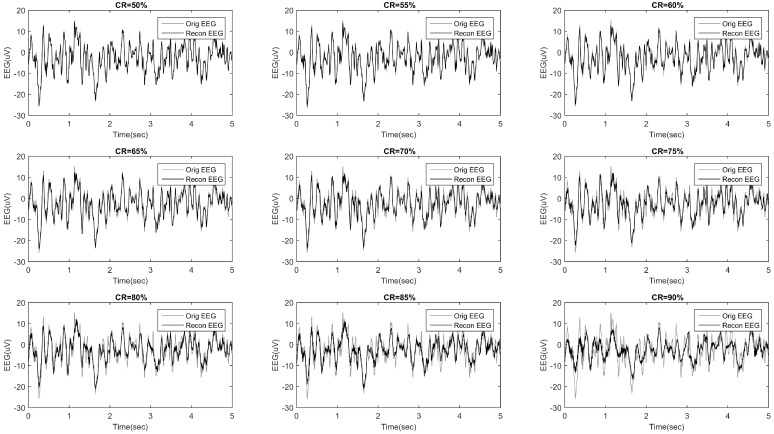
The original EEG recorded at channel E2 (**gray**) and the related compressively sensed signals (**black**) reconstructed at different compression rates (CR).

**Figure 4:**
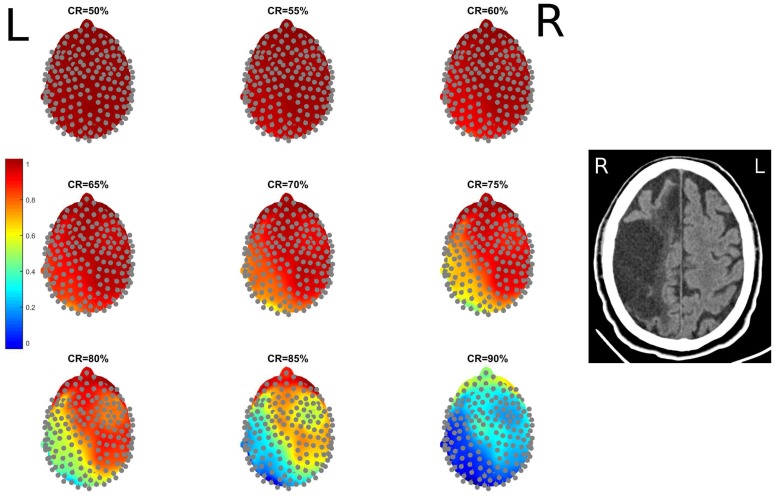
Topographical representation of the reconstruction quality of EEG signals, at increasing compression rates (CR). The SSIM values of Every EEG channels are depicted with a coloration ranging from blue (low SSIM) to red (high SSIM). Right (**R**) and Left (**L**) sides are indicated with black capital letters. The patient in this example is impaired in the right hemisphere and exhibits higher SSIM in the right hemisphere for every CR. The computed tomography (CT) image of the patient is shown on the right, Right (**R**) and Left (**L**) sides are indicated with white capital letters (note that Right and Left look reversed in CT images).

The methodology described in Section 2.3 and Section 2.4 was implemented and applied to the HD-EEG recordings described in Section 2.2. Figure 3 shows an example of an EEG signal compressed and then reconstructed at increasing CR (ranging from 50% to 90% with a 5% step). For CR = 50%, and even up to 80%, a very good match can be observed. Figure 4 shows an example of the topographical representation (mapping over the scalp) of the EEG reconstruction quality, for increasing CR. Given a compression rate CR and an epoch *e*, every EEG signal *x* is compressed and reconstructed and the related $SSIM^{e}\left(x\right)$ is estimated. The $SSIM^{e}\left(x\right)$ values are then averaged over the epochs to come up with a mean reconstruction quality SSIM(x) of channel *x* at the fixed compression rate. SSIM(x) values of the EEG channels are depicted in Figure 4 with a coloration ranging from blue (low SSIM) to red (high SSIM). Each map is associated to a different CR value. The patient in this example, Pt 13, has a stroke lesion in the right hemisphere (her/his RX image is shown in Figure 4) and exhibited higher SSIM in the right hemisphere than in the left one, for every CR. It is worth highlighting that, even for high CR values, the EEGs of the impaired hemisphere could be reconstructed with a higher quality (higher SSIM). Even at high levels of compression (CR = 80–85%), the hemisphere with lesion (right) showed a SSIM close to 1 (red). Even for CR = 90%, the hemisphere with the lesion still has relatively higher SSIM values than the healthy hemisphere. This result is likely due to the slowing effect, typically observed in the EEG of stroke patients, which is induced by the alterations caused by the lesion and makes EEG signals more regular [24,34,35,36].

### 3.1. Epoch-Based SSIM Comparison

Table 2 shows the results of the statistical comparison (described in Section 2.4.1) between the two vectors ${\mathrm{SSIM}}_{R}$ and ${\mathrm{SSIM}}_{L}$, for each patient and for every CR, when the high-density electrode montage was used. The results show that the reconstruction quality of EEG signals reflects cerebral asymmetries; the median SSIM was indeed larger in the impaired hemisphere than in the healthy one (*p* < 0.05, significant p values are highlighted in bold in Table 2), for at least seven CR values out of nine, except in patients Pt 10 and Pt 12, who exhibited such a behavior at four CR values out of nine. The same procedure was carried out selecting only the electrodes included in the standard Low-Density EEG montage (black electrodes in Figure 1b). From the 162 scalp electrodes included in the present study, the electrodes corresponding to standard low-density 10–20 montage were selected, namely Fp1, Fp2, F7, F3, F4, F8, T3, C3, C4, T4, T5, P3, P4, T6, O1, O2, excluding the electrodes on the middle line (Fz, Cz and Pz). Such channels were divided into two sub-sets: the right and left hemisphere (odd numbers are left electrodes and even numbers are right electrodes) and the epoch-based analysis described in the present section was repeated. Table 3 shows the results of the statistical analysis when the standard low-density montage was used. The median SSIM were not larger in the impaired hemisphere than in the healthy one (*p* > 0.05), thus no significant difference between the reconstruction quality of the EEG signals of the two hemispheres could be observed.

### 3.2. Overall SSIM Comparison

**Figure 5:**
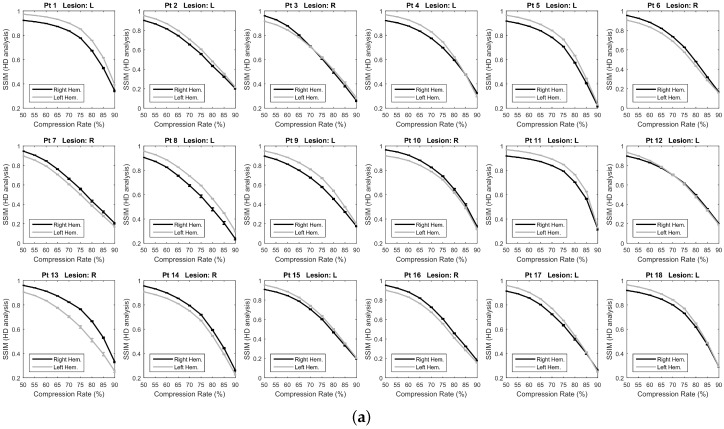

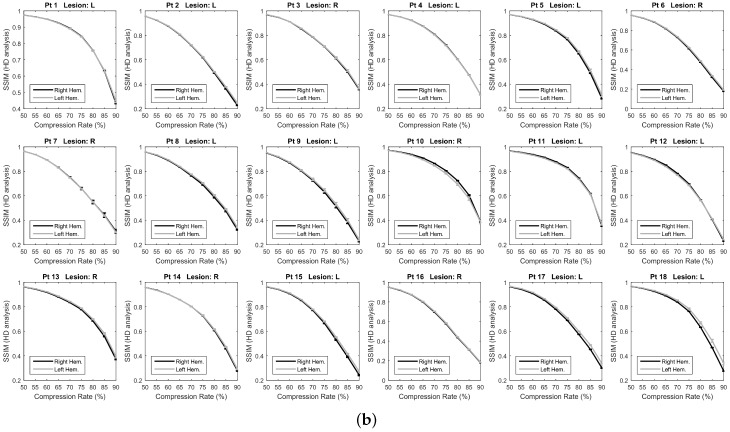
Comparison of the reconstruction quality of the EEG signals of the right (black line) and left (gray line) hemispheres. The average SSIM of the two hemispheres is depicted for increasing Compression Rates (CR). The analysis was carried out using: (**a**) High-Density EEG montage; and (**b**) Low-Density EEG montage.

**Figure 6:**
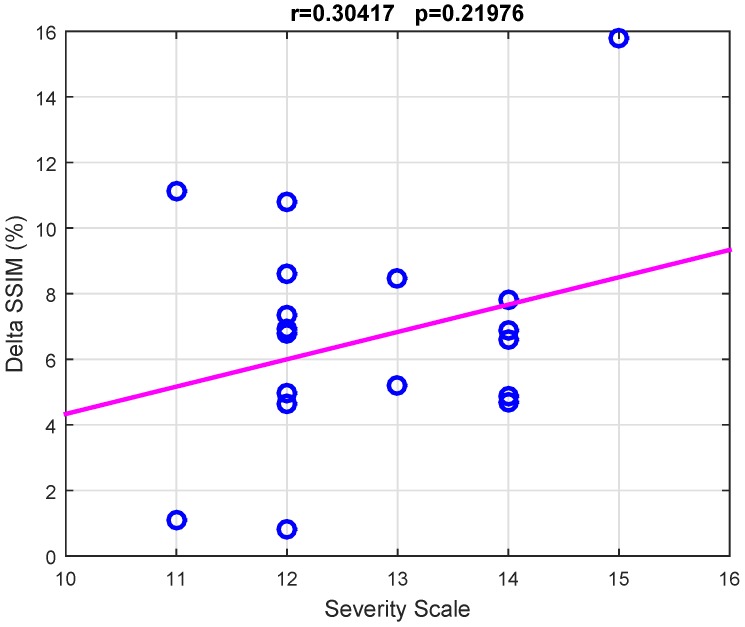
Analysis of the correlation between the severity scale and differences in the compressibility of the EEG signals of the two hemispheres. The correlation between ΔSSIM and the severity scale was assessed by the Pearson’s linear correlation test. Each blue circle represents the severity scale vs. ΔSSIM of a given subject. Since p=0.21976, no significant correlation between ΔSSIM and the severity scale could be observed.

**Figure 7:**
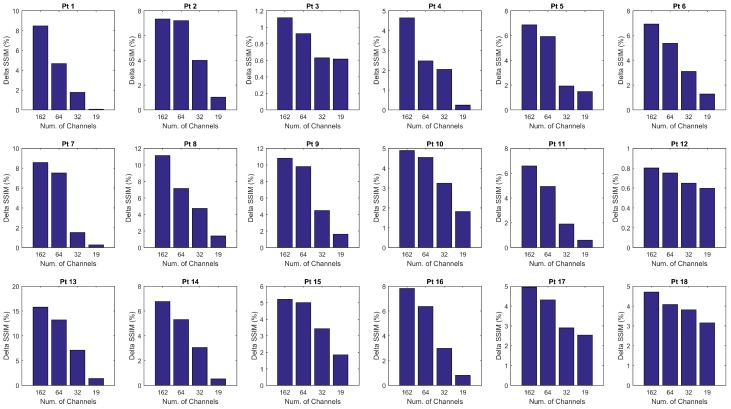
Effect of the number of channels on the estimated overall compressibility of the two hemispheres. For every patient, the percent difference between the areas under the two SSIM curves shown in Figure 5, denoted as ΔSSIM, was calculated with 19, 32, 64 and 162 channels, all subsampled from 256 channels.

As described in Section 2.4.2, for each patient, the mean SSIM of the two hemispheres (${\bar{SSIM}}_{R}$ and ${\bar{SSIM}}_{L}$) were also estimated by averaging the values $SSIM^{e}{\left(x\right)}_{R}$ and $SSIM^{e}{\left(x\right)}_{L}$ over the channels *x* and over the epochs *e* of the given hemisphere. In this way, the SSIM vs. CR trend displayed in Figure 5a was obtained. The SSIM curve of the impaired hemisphere stands above the curve of the healthy hemisphere for every CR, which means that the EEGs recorded at the lesioned hemisphere hold a higher compressibility than EEGs recorded at the healthy hemisphere. The lesion induced an increased regularity in the signals, probably because of the slowing effect. An odd behavior can be observed in Patients 3 and 12 as the two curves look overlapped except at lower compression rates. A reason for that could be that the lesion did not greatly affect the regularity of EEG signals, thus making SSIM analysis not very sensitive to CR. The slight difference between the two SSIM curves that is visible at low CR might also be due to the effect of averaging over the channels. The procedure described in Section 2.4.2 was also applied to a spatially subsampled dataset, including in the analysis only the electrodes belonging to the low-density EEG montage (black electrodes in Figure 1b). The average SSIM of the right hemisphere (${\bar{SSIM}}_{R}$) and of left hemisphere (${\bar{SSIM}}_{L}$) was estimated, as plotted in Figure 5b. As can be observed, the two SSIM curves look overlapped, thus low-density analysis did not allow to detect significant differences in the compressibility of the EEG signals of the two hemispheres. Despite the redundancy associated with the high number of EEG channels and linked to the volume conduction phenomena, high-density EEG has allowed better describing the properties of cerebral electrical activity, with particular reference to the compressibility and to its relationship with abnormalities caused by stroke lesions. To quantify the results observed in Figure 5, the percent difference between the areas under the two right and left SSIM curves was calculated and is denoted as ΔSSIM. Possible relationships between the severity scale and differences in the compressibility of the EEG signals of the two hemispheres were assessed by studying the correlation between ΔSSIM and the severity scale (scored according to the NIH Stroke Scale [25]), by means of the Pearson’s linear correlation test. Figure 6 shows the scatter plot of severity scale vs. ΔSSIM; each blue circle represents a subject. The correlation was 0.30417 and the *p* value was 0.21976, therefore no significant correlation between ΔSSIM and the severity scale could be observed. In the authors’ opinion, a correlation could be hypothesized between ΔSSIM and the volume of lesions. In the present work, volume lesion load was not performed because of lesion sites’ heterogeneity. In addition, some patients underwent Computed Tomography (CT) examinations while others Magnetic Resonance Imaging (MRI) scans; these two radiological methods are not comparable in terms of volume load findings and were used in the present work only to assess what hemisphere the lesion was localized in. In future studies, all patients will undergo MRI and the achieved results will be correlated with the volume of lesions. To investigate how sensitive is the method to electrode density, further investigations were carried out estimating ΔSSIM when intermediate montages are adopted. In particular, ΔSSIM was calculated with 19, 32, 64 and 162 channels, all subsampled from 256 channels, at CR=50%. The configurations with 32 and 64 electrodes followed the standard 10–20 EEG montages with 32 and 64 channels. Figure 7 shows the achieved ΔSSIM vs. the adopted montage, for every patient. The configuration with 162 channels resulted associated with the highest ΔSSIM, therefore it was able to detect asymmetries in the compressibility of the electrical activity of the two hemispheres more than standard montages with 19, 32, snf 64 channels.

## 4. Discussion

Stroke is a phenomenon that can be caused by a hemorrhagic or ischemic event that alters neuronal connections [35]. There is increasing evidence that, following the critical event, the brain tries to reshape itself in order to establish compensatory neural connections [37]. Such reorganization unavoidably reflects upon the brain electrical activity. The aim of the present research was to quantify the effects of the presence of the lesion on the cerebral electrical activity. In the future, objective parameters to score the efficacy of a treatment may be based on such quantification. In particular, the main objective of the present work was to evaluate possible asymmetries in the cerebral electrical activity caused by the presence of a lesion due to stroke in one of the two hemispheres. Specifically, the objective was to investigate the effects of the presence of a lesions on the compressibility of High-Density EEG (HD-EEG) signals recorded at the impaired hemisphere. The secondary objective was to investigate whether, using the standard 10/20 low-density electrode montage, such asymmetries between the two hemispheres could be detected. For this purpose, a group of 18 patients with unilateral stroke was recruited at IRCCS Centro Neurolesi Bonino-Pulejo (Messina, Italy). The patients underwent HD-EEG recording as well as neuroradiological evaluations to localize the lesion. The compression and subsequent reconstruction of each HD-EEG signal was performed using Compressive Sensing based on Block-Sparse Bayesian Learning [19]. The quality of the reconstruction, measured through SSIM at increasing compression rate values, allowed to detect differences in the compressibility of the EEG recorded at the two hemispheres. The injured hemisphere resulted associated with a higher compressibility of EEG signals, which indeed exhibited higher SSIM (*p* < 0.05) than those recorded at the healthy hemisphere. This result is likely due to the higher regularity of the signals caused by the slowing effect induced by the presence of lesions. The slowdown is a sign of suffering, in the areas involved in the lesion, which is due to alterations of the intracerebral connections (alteration caused by the reduction of oxygen and metabolites) in terms of both number of connections and speed of communication between neurons. In addition, the affected area unbalances the intra-hemispheric ratio and, therefore, the healthy hemisphere becomes predominant compared to the impaired one [24,34,35,36]. The analysis conducted using the standard low-density EEG montage did not allow detecting such differences between the two hemispheres. It seems that low-density EEG cannot cover the region of the lesion with a spatial resolution that is high enough to capture differences in the compressibility of the two hemispheres and that HD-EEG allows describing in more detail the asymmetry in the electrical brain activity related to the presence of lesions in stroke patients. The proposed method could be particularly useful in the patient’s follow-up, to evaluate the ability of the ongoing treatments to help restoring symmetry between the two hemispheres. In the future, a HD-EEG based neurofeedback system will be developed for active patient rehabilitation and, given the promising results achieved with the presented compressive sensing analysis, the compressibility of EEG signals could be one of the involved parameters. Even though standard low-density EEG is faster, cheaper and more widespread than HD-EEG and quantitative EEG (qEEG) is able to provide an estimate of EEG asymmetries, the future aim is to combine the method proposed in the present work with source reconstruction to study the compressibility of the brain electrical activity at source level. The use of HD-EEG will be necessary, as it was shown to allow for a far more precise source reconstruction than standard EEG [38]. The in-depth analysis of the compressibility of the electrical activity of the brain at source level will allow understanding how intact areas in the brain differ from the lesioned ones. The study will also be detailed in the different EEG sub-bands (delta, theta, alpha, beta, and gamma) and will be extended to a larger number of patients. Patients will be evaluated longitudinally to quantify the effects of treatment throughout the follow-up program. The effectiveness of the treatment is expected to reflect upon the asymmetry of the SSIM of the two hemispheres, which is indeed expected to decrease as the treatment helps to balance their functionality. If so, measures of reconstruction quality could be used in the future as a biomarker for the longitudinal evaluation of the efficacy of the treatment.

## 5. Conclusions

Research shows that, following a stroke, neuronal connections are disrupted. The main objective of the present work was to evaluate possible asymmetries in the cerebral electrical activity caused by the presence of a lesion due to stroke. Specifically, the main objective was to study the compressibility of the EEG signals recorded at the two hemispheres to assess whether the presence of lesions in one of them caused any asymmetry. Eighteen subjects with unilateral stroke were recruited and monitored through High-Density (HD-EEG). The secondary objective of the present research was to investigate whether the hypothesized asymmetry in the electrical activity of the two hemispheres could be detected using only the channels of the standard low-density montage. Given a patient, each EEG signal was processed by means of compressive sensing and then reconstructed, at increasing compression rates. The quality of the reconstruction, measured through the SSIM, showed differences in the compressibility of the impaired and healthy hemispheres. The EEG signals recorded at the impaired hemisphere exhibited a higher reconstruction quality (higher SSIM) compared to the EEG signals recorded at the healthy hemisphere (*p* < 0.05), for every compression rate. This is likely related to the slowing effects caused by the lesion and, therefore, to the increased regularity of EEG signals. The analysis, when conducted using only the standard low-density EEG channels, did not allow detecting significant differences between the two hemispheres (*p* > 0.05). The compressibility analysis of HD-EEG seems to be able to describe the asymmetry in the electrical activity of the brain caused by the presence of stroke lesions.

## Figures and Tables

**Table 1:** Demographics.

Patient ID	AGE	GEN	Sev. Scale	Stroke Type	Stroke Site	Lesion Site
Pt 1	53	M	13	Ischemic	Silvian artery, Fronto-Temporal-Parietal Areas	Left
Pt 2	37	M	12	Hemorrhagic	Pontine hemorrhage	Left
Pt 3	27	M	11	Hemorrhagic	Frontal Lobe	Right
Pt 4	73	F	12	Ischemic	Silvian artery, Fronto-Temporal-Parietal Areas	Left
Pt 5	86	M	14	Ischemic	Thalamus, Posterior limb internal capsule	Left
Pt 6	61	F	12	Hemorrhagic	Frontal Lobe	Right
Pt 7	76	M	12	Ischemic	Periventricular and cortical white matter lesions	Right
Pt 8	59	M	11	Hemorrhagic	Posterior limb internal capsule	Left
Pt 9	66	M	12	Hemorrhagic	Anterior cerebral artery and Frontal lobe	Left
Pt 10	72	M	14	Ischemic	Pontine	Right
Pt 11	81	M	14	Ischemic	Silvian artery, Fronto-Temporal-Parietal Areas	Left
Pt 12	66	M	12	Hemorrhagic	Pontine hemorrhage	Left
Pt 13	76	M	15	Ischemic	Complete middle cerebral artery stroke	Right
Pt 14	72	M	12	Hemorrhagic	Thalamus, Posterior limb internal capsule	Right
Pt 15	71	M	13	Ischemic	Pontine	Left
Pt 16	55	M	14	Hemorrhagic	Thalamus, Posterior limb internal capsule	Right
Pt 17	72	M	12	Hemorrhagic	Cerebellum	Left
Pt 18	79	M	14	Hemorrhagic	Frontal-Temporal-Parietal areas	Left

**Table 2:** Statistical comparison (*p* values) of the SSIM of the two hemispheres (analysis of High-Density EEGs).

Patient	CR = 50%	CR = 55%	CR = 60%	CR = 65%	CR = 70%	CR = 75%	CR = 80%	CR = 85%	CR = 90%
**Pt 1**	4.41E-28	7.68E-28	2.93E-25	1.95E-22	9.12E-21	1.01E-19	1.25E-15	2.05E-10	7.62E-05
**Pt 2**	3.12E-23	1.72E-16	3.96E-11	6.11E-07	1.20E-05	2.51E-04	7.90E-04	1.53E-02	1.91E-01
**Pt 3**	9.50E-20	1.78E-05	2.68E-01	3.15E-01	3.66E-02	7.67E-03	1.20E-02	1.56E-02	8.30E-03
**Pt 4**	7.68E-28	2.52E-24	1.34E-21	1.77E-16	1.12E-11	2.82E-07	9.66E-02	1.06E-01	8.66E-08
**Pt 5**	4.63E-27	2.50E-20	1.30E-13	1.31E-09	1.59E-08	2.64E-08	6.82E-10	9.95E-06	1.68E-01
**Pt 6**	5.50E-28	2.80E-24	3.87E-20	5.07E-16	9.91E-11	6.40E-10	3.32E-08	4.45E-06	6.90E-01
**Pt 7**	1.93E-15	6.67E-08	1.51E-05	1.85E-04	1.14E-03	1.86E-03	1.41E-02	1.79E-02	1.85E-02
**Pt 8**	1.67E-25	6.96E-18	5.16E-09	7.17E-06	9.65E-05	1.71E-04	2.03E-04	8.76E-05	3.73E-05
**Pt 9**	2.73E-25	2.61E-19	3.78E-15	1.26E-12	1.44E-10	7.27E-10	1.94E-08	5.43E-05	6.08E-03
**Pt 10**	1.79E-27	2.46E-19	1.98E-08	3.53E-03	1.68E-01	4.88E-01	5.35E-01	6.75E-01	7.03E-01
**Pt 11**	6.62E-28	5.89E-25	2.01E-22	2.17E-19	1.87E-16	1.71E-14	7.25E-16	6.06E-14	2.72E-03
**Pt 12**	8.25E-06	1.39E-01	8.88E-01	4.12E-01	2.96E-01	1.15E-01	2.42E-02	2.61E-02	4.10E-03
**Pt 13**	2.19E-26	7.71E-23	2.17E-19	2.54E-16	2.22E-14	1.12E-14	3.71E-13	1.32E-10	2.14E-06
**Pt 14**	8.21E-26	6.15E-16	1.53E-07	2.32E-04	2.20E-03	9.34E-04	6.26E-04	7.20E-05	1.88E-03
**Pt 15**	6.76E-18	7.74E-08	8.40E-05	3.45E-03	3.66E-02	8.69E-02	1.01E-01	4.82E-01	9.39E-01
**Pt 16**	7.42E-27	2.24E-19	3.81E-13	6.43E-09	9.26E-05	6.71E-05	1.46E-05	1.21E-06	6.26E-05
**Pt 17**	1.77E-22	1.78E-15	3.37E-10	8.50E-08	5.43E-05	1.31E-02	2.32E-01	8.70E-01	1.61E-01
**Pt 18**	2.92E-26	2.10E-17	6.41E-12	2.26E-08	4.90E-06	2.58E-04	5.71E-02	8.99E-01	3.91E-01

**Table 3:** Statistical comparison (*p* values) of the SSIM of the two hemispheres (analysis of Low-Density EEGs).

Patient	CR = 50%	CR = 55%	CR = 60%	CR = 65%	CR = 70%	CR = 75%	CR = 80%	CR = 85%	CR = 90%
**Pt 1**	6.45E-01	9.59E-01	6.45E-01	5.74E-01	4.42E-01	4.42E-01	7.98E-01	7.98E-01	8.78E-01
**Pt 2**	7.98E-01	1.00E+00	9.59E-01	8.78E-01	8.78E-01	8.78E-01	5.74E-01	4.42E-01	3.28E-01
**Pt 3**	3.82E-01	5.05E-01	7.98E-01	7.21E-01	8.78E-01	7.21E-01	6.45E-01	7.98E-01	4.42E-01
**Pt 4**	8.78E-01	5.74E-01	5.74E-01	9.59E-01	9.59E-01	9.59E-01	6.45E-01	7.21E-01	9.59E-01
**Pt 5**	9.59E-01	6.45E-01	6.45E-01	8.78E-01	1.00E+00	8.78E-01	7.21E-01	7.21E-01	9.59E-01
**Pt 6**	6.45E-01	7.21E-01	7.98E-01	5.05E-01	5.05E-01	3.82E-01	3.82E-01	1.61E-01	1.61E-01
**Pt 7**	9.59E-01	9.59E-01	7.21E-01	9.59E-01	9.59E-01	8.78E-01	7.98E-01	6.45E-01	6.45E-01
**Pt 8**	9.59E-01	5.05E-01	7.21E-01	6.45E-01	6.45E-01	7.98E-01	6.45E-01	8.78E-01	6.45E-01
**Pt 9**	5.74E-01	5.74E-01	5.74E-01	5.74E-01	5.74E-01	5.74E-01	5.05E-01	7.98E-01	9.59E-01
**Pt 10**	7.98E-01	7.98E-01	5.74E-01	7.21E-01	7.21E-01	8.78E-01	7.21E-01	7.21E-01	9.59E-01
**Pt 11**	2.34E-01	2.79E-01	2.34E-01	2.34E-01	2.34E-01	4.42E-01	3.28E-01	5.74E-01	6.45E-01
**Pt 12**	1.61E-01	1.95E-01	2.34E-01	1.95E-01	3.82E-01	7.21E-01	7.98E-01	8.78E-01	9.59E-01
**Pt 13**	1.00E+00	6.45E-01	5.74E-01	4.42E-01	3.82E-01	4.42E-01	5.74E-01	8.78E-01	9.59E-01
**Pt 14**	1.00E+00	1.00E+00	8.78E-01	8.78E-01	9.59E-01	5.74E-01	5.74E-01	3.28E-01	5.74E-01
**Pt 15**	5.05E-01	6.45E-01	4.42E-01	3.28E-01	7.21E-01	3.82E-01	3.82E-01	5.05E-01	3.82E-01
**Pt 16**	4.42E-01	7.21E-01	9.59E-01	7.98E-01	9.59E-01	1.00E+00	8.78E-01	9.59E-01	9.59E-01
**Pt 17**	3.28E-01	2.34E-01	1.61E-01	1.05E-01	1.05E-01	1.61E-01	1.95E-01	8.30E-02	8.30E-02
**Pt 18**	5.74E-01	4.42E-01	1.95E-01	1.61E-01	1.05E-01	1.30E-01	1.95E-01	2.34E-01	1.61E-01
